# Glutaric Acid-Mediated Apoptosis in Primary Striatal Neurons

**DOI:** 10.1155/2014/484731

**Published:** 2014-05-12

**Authors:** Fengyan Tian, Xi Fu, Jinzhi Gao, Yanqin Ying, Ling Hou, Yan Liang, Qin Ning, Xiaoping Luo

**Affiliations:** ^1^Department of Pediatrics, Tongji Hospital of Tongji Medical College, Huazhong University of Science and Technology, Wuhan, Hubei 430030, China; ^2^Department of Pediatrics, First Affiliated Hospital of Zhengzhou University, Zhengzhou, Henan 450052, China; ^3^Laboratory of Infectious Immunology, Department of Infectious Disease, Tongji Hospital of Tongji Medical College, Huazhong University of Science and Technology, Wuhan, Hubei 430030, China

## Abstract

Glutaric acid (GA) has been implicated in the mechanism of neurodegeneration in glutaric aciduria type I. In the present study, the potential cytotoxic effects of GA (0.1~50 mM for 24~96 h) were examined in cultured primary rat striatal neurons. Results showed increase in the number of cells labeled by annexin-V or with apoptotic features shown by Hoechst/PI staining and transmission electron microscopy (TEM) and upregulation of the expression of mRNA as well as the active protein fragments caspase 3, suggesting involvement of the caspase 3-dependent apoptotic pathway in GA-induced striatal neuronal death. This effect was in part suppressed by the N-methyl-D-aspartate (NMDA) receptor antagonist MK-801 but not the **α**-amino-3-hydroxy-5-methylisoxazole-4-propionic acid (AMPA) antagonist 6-cyano-7-nitroquinoxalone-2,3-dione (CNQX). Thus, GA may trigger neuronal damage partially through apoptotic pathway and via activation of NMDA receptors in cultured primary striatal neurons.

## 1. Introduction

Glutaric aciduria type I (GA I) is an autosomal recessive organic aciduria caused by mutations in the gene encoding glutaryl-CoA dehydrogenase (GCDH). Patients are at risk of developing an irreversible dystonia and/or dyskinesia movement disorders as a sequence of selective destruction of striatal neurons following acute encephalopathic crises triggered by intercurrent catabolic situations such as infectious diseases, fever, vomiting, diarrhea, or immunization. The acute episodes of encephalopathy usually occurred within the first 3 years of life [1]. The main pathomorphological findings are frontotemporal atrophy, basal ganglia lesions, white matter disease, and postsynaptic vacuolization of neurons [2]. However, the clinical phenotype has no relationship to the biochemical phenotype or the genotype [1, 3].

The pathomechanism of maturation- and region-dependent neurotoxicity has not been established. Several model systems have been used to study the neuropathology in GA I. Animal models and* in vivo* studies are listed as follows. The fruit-eating bat* Rousettus aegyptiacus*, a natural animal model for GA I, which lacks hepatic and renal GCDH activity but retains cerebral GCDH activity, presents similar biochemical phenotype but lacks neurologic manifestations typical in GA I patients [4]. “Chemical” rat model, produced by intrastriatal/intracerebroventricular administration or subcutaneous/intraperitoneal injection with GA [5, 6], fails to duplicate pathophysiological situation in patients owing to the single dose administration as well as possessing normal GCDH activity. The hepatic* Gcdh* ^−*/*−^ mice also only have similar biochemical phenotype whereas the cerebral concentrations of GA-related metabolites and the neurological manifestations are normal [7]. The knock out (KO) mouse model with complete loss of GCDH activity, reproduced biochemical phenotype but its clinical and striatal pathologic features are not corresponding to GA I patients: without typical encephalopathic crises triggered by catabolic state as well as without striatal neuronal loss and gliosis thus correlating with the absence of progressive dystonia but only presenting a mild motor deficit [8, 9]. Although the diet-induced 4-week-old* Gcdh* ^−*/*−^ mice not the 8-week old mice provoked a phenotype similar to encephalopathic crisis, they died within 3–6 days of lysine diet exposure [10]. Furthermore, macrocephaly is present at or shortly after birth in many patients and the neurological manifestations usually are apparent within the first 3 years of life, matching to mice within approximately the first 15 days of life [11], so the adoption of 4-week-old KO mice is also inappropriate due to the discordance with the susceptible age of patients.* In vitro* studies include primary neuronal cells from chick, rat, wildtype, or* Gcdh* ^−/−^ mice brain [12], mixed neuronal and glial cultures, astrocytic cultures, rat immature oligodendroglia cell line [13], synaptic plasma membranes [14], brain homogenates from rats [15], organotypic slice cultures, and rat 3D organotypic brain cell cultures in aggregates [16], aiming at studying the neurotoxic effects on nerve cells. Human dermal microvascular endothelial cells, HEK293 cells, CHO cells,* Xenopus* oocytes, human choroid plexus epithelial cells, and porcine brain capillary endothelial cells (pBCEC) were used to study the transport of GA metabolites and the role of blood-brain barrier (BBB) [17]. These* in vitro* models are useful to investigate the separate contribution of single metabolite to the specific cell or tissue damage in GA-I. Children with GA I suffer selective striatal degeneration with severe neuronal loss. Thus, in the present study, primary striatal neurons were used to assess the possible striatal neuronal damage mechanism triggered by GA.

## 2. Materials and Methods

### 2.1. Materials

The following chemicals were used: GA, MK-801, CNQX, poly-l-lysine (PLL), Hoechst 33342, propidium iodide (PI) (Sigma-Aldrich Chemical Co., St. Louis, MO), 3-(4,5-dimethylthiazol-2-yl)-2,5-diphenyltetrazolium bromide (MTT), dimethyl sulfoxide (DMSO) (Amresco Inc., Solon, OH), Dulbecco's minimum essential medium- (DMEM-) high glucose, Neurobasal (NB) medium, B27, trypsinase, fetal bovine serum (FBS), horse serum (HS) (Gibco Company, Grand Island, NY), annexin V-FITC (Jingmei Biotech, Beijing, China), TRIZOL reagent (Invitrogen Co., Carlsbad, CA), RevertAid First Strand cDNA synthesis kit (Fermentas Life Sciences, Vilnius, Lithuania), SYBR Premix Ex Taq^II^ (Takara Biotechnology, Dalian, China), M-PER Mammalian Protein Extraction Reagent, Pierce BCA Protein Assay Kit and SuperSignal West Pico Chemiluminescent Substrate (Thermo Scientific, Rockford, IL), rabbit polyclonal antibody to caspase 3 and *β*-actin (Cell Signaling Technology, Beverly, MA). HRP-goat anti-rabbit IgG (H+L) conjugate was obtained from Invitrogen.

### 2.2. Cell Culture

Sprague-Dawley (SD) rats (SPF grade) were purchased from the Laboratory Animal Center, Tongji Medical College. Cultures of primary striatal neurons were prepared from the brains of neonatal SD rats (within 24 h of birth) [18]. After decapitation and removal of the brains, the corpora striata were separated from the brain, washed three times, and preserved in ice-cold HBSS without calcium and magnesium. The corpora striata were minced and then digested in trypsin (0.125%) for 15 min at 37°C. Following the addition of the seeding medium (DMEM-high glucose medium supplemented with 10% FBS, 10% HS, and 2 mM L-glutamine) to terminate the digestion, cells were dispersed by repeated pipetting and filtration through a 200-mesh cell strainer, suspended, and seeded at a density of 1 × 10^6^/mL on poly-L-lysine-coated 96- or 6-well plates. On the 2nd day, medium was replaced with serum-free neurobasal medium containing 2% B27 and 0.5 mM L-glutamine (i.e., feeding medium). Cells were maintained in the feeding medium, which was replaced every second day for up to 10**~**14 days, and incubated in a humidified atmosphere with 5% CO_2_ at 37°C. The purity of neuronal cultures was determined by neuron-specific enolase (NSE) immunocytochemistry performed on days* in vitro *(DIV) 7. Experiments were conducted on rat striatal neurons in culture for 10~14 days.

### 2.3. Treatments and Groups

Neurons (DIV 10) were submitted to GA (0~50 mM, 24**~**96 h) with or without pre- (15 min) and coapplication of 10 *μ*M MK-801 or CNQX. MK-801 is a NMDA receptor antagonist while CNQX is a non-NMDA receptor (AMPA receptor) antagonist. GA was dissolved in the feeding medium (0.1 M as a stock concentration) while the antagonists were dissolved in 0.01 M PBS (1 mM as a stock solution). All solutions were adjusted to pH 7.2**~**7.4.

### 2.4. Evaluation of Neuronal Viability (Mitochondrial Activity)

The functional status of intact mitochondria was assessed by evaluating cell viability using MTT [18]. Mitochondria of living cells, but not of dead cells, can convert yellow MTT into violet formazan salt. The amount of formazan generated is directly proportional to the cell number over a wide range. MTT (10 *μ*L of 5 mg/mL in 0.01 M PBS, sterile filtered; final concentration 0.5 mg/mL) was added to wells containing cells seeded in 96-well plates (50,000**~**100,000 cells/well), maintained for 10 d, and treated with various concentrations of GA for another 24, 48, 72, and 96 h. After incubation with MTT at 37°C for 4 h, the formazan was dissolved by addition of 150 *μ*L of DMSO. Absorbance was read on a Bio-Rad Model 680 microplate reader at a test wavelength of 570 nm and a reference wavelength of 630 nm.

### 2.5. Assessment of Cellular Morphology

Cells were incubated with GA as indicated above, stained with Hoechst 33342 (10 *μ*g/mL; 1 mg/mL stock solution in 0.01 M PBS) at 37°C for 10 min then with PI (10 *μ*g/mL; 1 mg/mL stock solution in 0.01 M PBS) at 4°C, fixed with 4% paraformaldehyde in PBS at 4°C for 10 min, and washed three times with 0.01 PBS. Changes in cellular morphology including the nuclear morphology were observed under an inverted fluorescence microscope and cells with nuclear condensation of chromatin and/or nuclear fragmentation were counted in 8 randomly chosen fields per group.

### 2.6. Detection of Apoptosis

Flow cytometry analysis of cells double-stained with FITC-Annexin V and PI according to the manufacturer's instructions was used to determine the ratio of apoptotic cells to total cells. In brief, cells treated with GA as above were collected, washed twice with cold PBS, and resuspended to a density of 1 × 10^6^ cells/mL in binding buffer (10 mM HEPES/NaOH, pH 7.4, 140 mM NaCl, and 2.5 mM CaCl_2_). Cell suspension (100 *μ*L) was incubated with 5 *μ*L of FITC-Annexin V and 10 *μ*L of PI (20 *μ*g/mL) at room temperature for 15 min, diluted with another 400 *μ*L of binding buffer, and analyzed using a flow cytometer (Becton Dickinson, USA) at 488 nm to distinguish living cells (Annexin V^−^/PI^−^) from early apoptotic cells (Annexin V^+^/PI^−^), late apoptotic cells (Annexin V^+^/PI^+^), and necrotic cells (Annexin V^−^/PI^+^).

### 2.7. Transmission Electron Microscopy

Cells were harvested, washed 3 times with 0.01 M PBS, and then fixed in 3% glutaraldehyde. Samples were postfixed with 1% OsO_4_ in sodium cacodylate trihydrate at room temperature, dehydrated in a graded series of ethanols, embedded, and then sectioned for transmission electron microscopy (TEM). The sections were examined under a Holland FEI Tecnai G2-12 transmission electron microscope.

### 2.8. Real-Time RT-PCR

Total RNA was extracted from striatal cells by TRIZOL reagent following the manufacturer's instructions. The quantity and purity of total RNA were determined by measuring absorbance at 260 nm and 280 nm, and the OD_260_/OD_280_ ratio was in the range of 1.8~2.0. The concentration of total RNA was determined from the absorbance at 260 nm. One *μ*g of total RNA was reversely transcribed into cDNA using a RevertAid First Strand cDNA Synthesis kit according to the manufacturer's instructions. The PCR primers (Table 1) were designed by Premier Primer 5.0 software and BLAST searched against all the nucleotide databases. All primers were synthesized by Invitrogen, Shanghai, China. One *μ*L of cDNA was amplified by quantitative PCR performed using SYBR Green Reagents and an ABI Prism 7500 Real-Time PCR system (Applied Biosystems, Darmstadt, Germany). As recommended by the manufacturer, cDNA amplification was performed in 40 cycles of denaturation (15 sec at 95°C), annealing (15 sec at 59**~**61°C), and primer extension (34 sec at 72°C). The fluorescent signals were collected during the extension phase, and Ct values of the samples were calculated and normalized to the house-keeping gene GAPDH. The specificity of the PCR products was determined by melting curve analysis. A negative PCR using water instead of cDNA was performed as a control. Each measurement was carried out in triplicate and repeated. The level of transcripts was measured using the comparative CT (2^−ΔΔCt^) method.

### 2.9. Immunoblot Analysis

Cells were harvested, washed once with ice-cold 0.01 M PBS, and lysed on ice using M-PER Mammalian protein extraction reagent according to the manufacturer's protocol. After removal of cell debris by centrifugation, the protein (concentration estimated using a Pierce BCA Protein Assay Kit) of the cell supernate (30 *μ*g) was fractionated by SDS-polyacrylamide gel electrophoresis (PAGE) and then transferred to PVDF membranes using a transfer system (Bio-Rad, Hercules, CA). The membranes were blocked with 5% nonfat dry milk for 1 h at room temperature, incubated with primary antibodies overnight at 4°C, washed, incubated with goat anti-rabbit IgG (H+L) HRP conjugate (1 : 5000) for 1 h, and developed using the SuperSignal West Pico Chemiluminescent Substrate. Representative Western blots are shown in the figures.

### 2.10. Statistical Analysis

Each experiment was repeated at least three times. Data are given as mean ± S.E.M. For statistical comparison, when the variances were equal in Levene's test for homogeneity of variance, one-way analysis of variance (ANOVA) followed by* post hoc* least significant difference (LSD) test was used; otherwise, Dunnett's T3 test was applied. Differences were considered significant at *P* < 0.05.

## 3. Results

### 3.1. Primary Culture and Identification of Striatal Neurons

**Figure 1:**
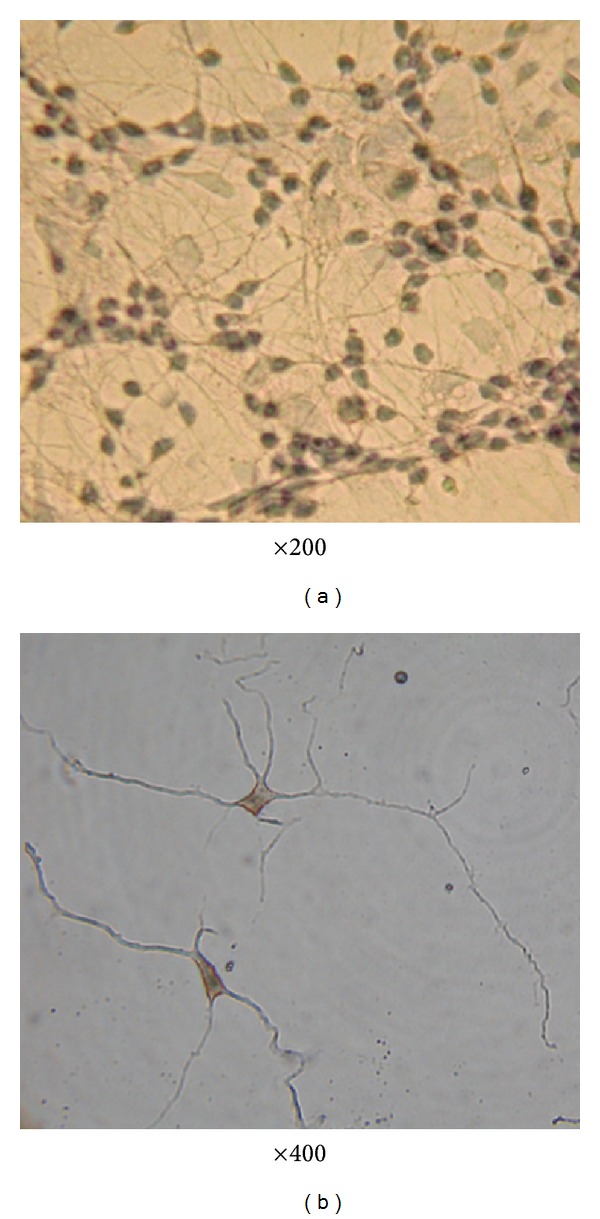
Neuron specific enolase immunocytochemistry of striatal neurons.

The purity of the primary striatal neuronal cells was assessed on the 7th day by immunoreactivity to neuron-specific enolase (NSE). In all, 91.37 ± 4.1% of the cells were NSE positive (Figure 1).

### 3.2. Cell Viability

**Figure 2:**
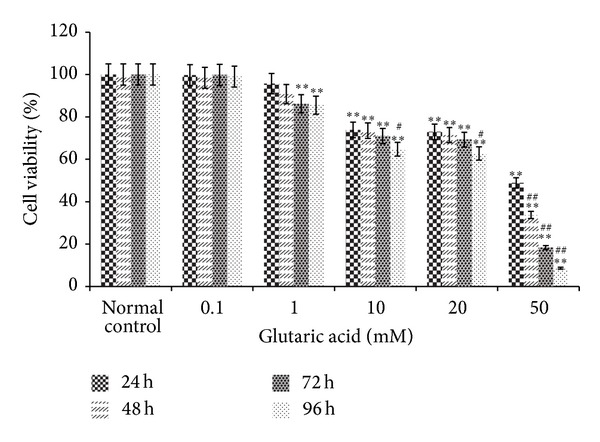
Viability of striatal cells as determined by MTT assay (OD ratio, mean ± SD, *n* = 6). *P < 0.05 and **P < 0.01 compared to control cultures incubated for the same amount of time; ^#^ *P* < 0.05 and ^##^ *P* < 0.01 compared to 24 h cultures exposed to the same GA concentration.

The effect of GA on neuron viability was measured after 24**~**96 h of exposure by the MTT test. Neuronal damage by GA was concentration- and time-dependent (Figure 2). Compared to cell viability in normal control sister cultures at the same time points of incubation, cell viability was significantly reduced in cultures treated with GA (10**~**50 mM) (*P* < 0.05) for 24, 48, 72, and 96 h or treated with 1 mM for 72 and 96 h. Neuronal survival (in %) was 86.18 ± 1.91, 85.52 ± 1.93 (*P* < 0.01, *n* = 6) at 72 and 96 h of incubation with 1 mM; 73.82 ± 1.83, 73.52 ± 3.22, 71.01 ± 1.44, 64.79 ± 2.38 (*P* < 0.01, *n* = 6) at 24, 48, 72, and 96 h incubation with 10 mM; 72.97 ± 1.51, 71.42 ± 4.68, 69.29 ± 1.35, 62.76 ± 2.41 (*P* < 0.01, *n* = 6; 20 mM), and 48.89 ± 1.24, 33.75 ± 1.02, 18.37 ± 5.82, 8.61 ± 4.49 (*P* < 0.01, *n* = 6; 50 mM) at 24, 48, 72, and 96 h of incubation with either 20 or 50 mM. After incubation with 50 mM for 48, 72, and 96 h, cell viability was decreased dramatically (*P* < 0.01) from its level at 24 h; after incubation with 10 and 20 mM for 96 h, it was also markedly decreased (*P* < 0.05) from its level at 24 h.

**Figure 3:**
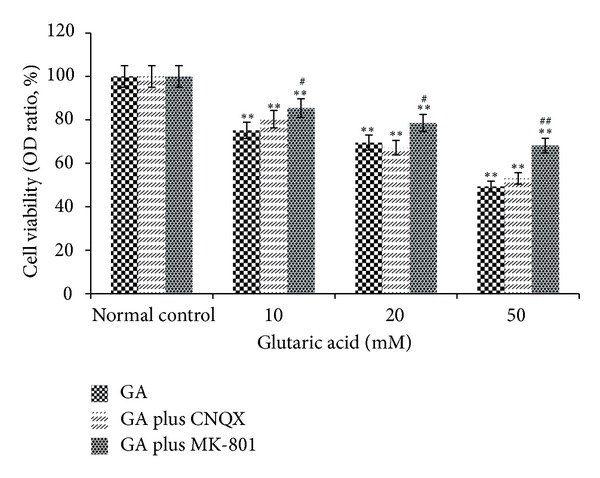
Viability of cultured striatal cells as determined by MTT assay after 24 h exposure to 10, 20, or 50 mM GA with or without pre- and coincubation with 10 *μ*M MK-801 or CNQX. *P < 0.05 and **P < 0.01, compared to control (0 mM GA with or without pre- and coincubation of the antagonist resp.); ^#^ *P* < 0.05 and ^##^ *P* < 0.01, cells with the pre- and coincubation of antagonist compared to cells treated with only GA at the same concentration.

To evaluate whether the toxic effect was mediated via the NMDA receptor, the ability of an NMDA receptor antagonist to alleviate GA-induced neurotoxicity was tested. The NMDA channel blocker MK-801 rather than CNQX significantly enhanced the survival of neurons treated with 10, 20, and 50 mM GA (*P* < 0.01) but failed to restore viability to its normal level (Figure 3).

### 3.3. Cellular Morphology

**Figure 4:**
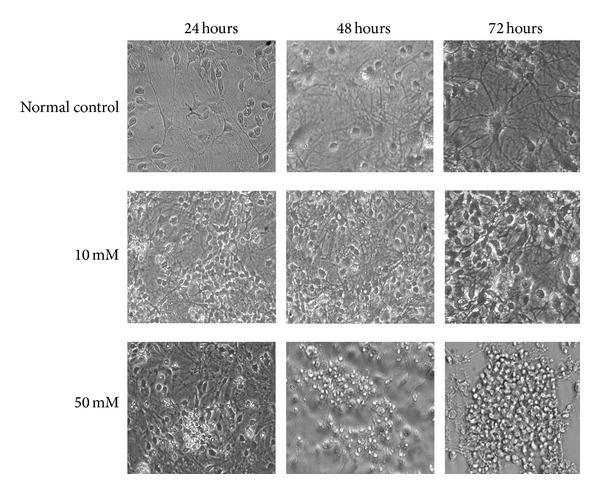
Morphological changes of striatal neurons in response to GA. Inverted microscopic image (×200) showing shrinkage, nuclear condensation, shortened dendrites, and cellular collapse.

**Figure 5:**
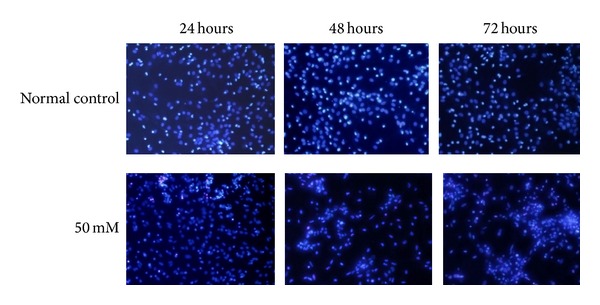
Nuclear changes revealed by Hoechst 33342/PI (×100). Apoptosis occurred in 30.0 ± 5.1% of the normal control cells and 65.3 ± 4.7%, 82.0 ± 5.4%, and 97.0 ± 2.9% of the cells treated with 50 mM (24, 48, and 72 h, resp.). Cells were shrunken and rounded and contained lunate or “horse-shoe” like nuclei (characteristic of apoptosis) with condensed chromatin.

Exposure to 50 mM GA for 24, 48, and 72 h resulted in obvious injury (Figures 4 and 5) with neuronal loss and degeneration increasing over time. Neurons typically appeared rounded and shrunken with karyopyknotic or karyolytic nuclei. Debris was visible instead of the neuronal network and neuronal bodies seen in cultures of normal control neurons. Hoechst staining showed nuclear condensation, shrinkage, and even collapse. Most of the cells were not obviously stained by PI.

### 3.4. Induction of Apoptosis

**Figure 6:**
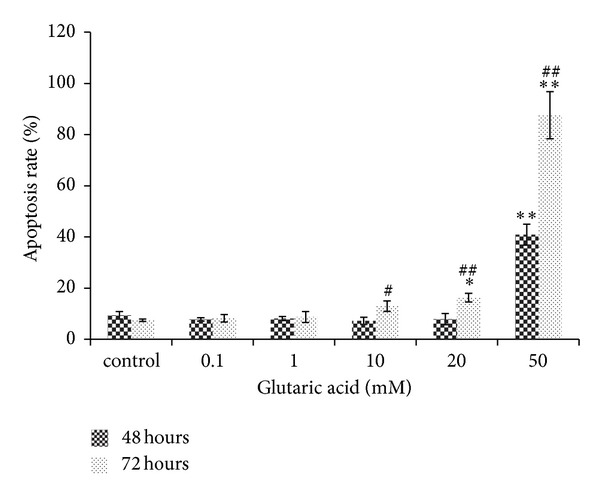
Detection of apoptotic/necrotic cells using Annexin V/PI staining. Note: *P < 0.05 and **P < 0.01, versus normal control cells incubated the same amount of time; ^#^ *P* < 0.05 and ^##^ *P* < 0.01, 72-h cells compared with 48-h cells at the same concentration.

**Figure 7:**
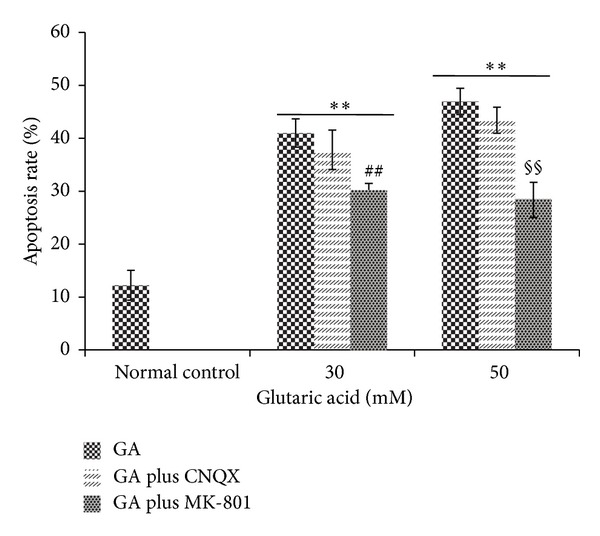
Rates of apoptosis in cells treated with 0, 30, and 50 mM GA for 24 h, in the presence or absence of 10 *μ*M MK-801 or CNQX, assayed by flow cytometry. *P < 0.05 and **P < 0.01, compared to normal control cells; ^#^ *P* < 0.05 and ^##^ *P* < 0.01, compared to cells treated with 30 mM GA and no antagonist. ^§^ *P* < 0.05 and ^§§^ *P* < 0.01, compared to cells treated with 50 mM GA and no antagonist.

Exposure of striatal neurons to GA led to a dose- and time-dependent increase in the number of apoptotic cells as assessed by flow cytometry (Figure 6). The increase in apoptosis rate was dose-dependent: 40.90 ± 4.09 (*P* < 0.01) and 87.63 ± 9.17 (*P* < 0.05), respectively, after exposure to 50 mM GA for 48 and 72 h and 16.27 ± 1.70 (*P* < 0.05) after exposure to 20 mM for 72 h. The increase was also time dependent: higher at 72 h than at 48 h for any given GA concentration (*P* < 0.05). The necrosis rate was comparable to that in the control. The NMDA receptor antagonist MK801 reduced the apoptosis rate from 41.00 ± 2.66 and 46.97 ± 2.47 (*P* < 0.01 for exposure to 30 mM and 50 mM GA for 24 h) to 30.23 ± 1.27 and 28.37 ± 3.32 (*P* < 0.01 for both), unlike the AMPA antagonist CNQX (37.83 ± 3.70, 43.40 ± 2.45; *P* > 0.05 for both), which had no effect (Figure 7).

### 3.5. Electron Microscopic Study

**Figure 8:**
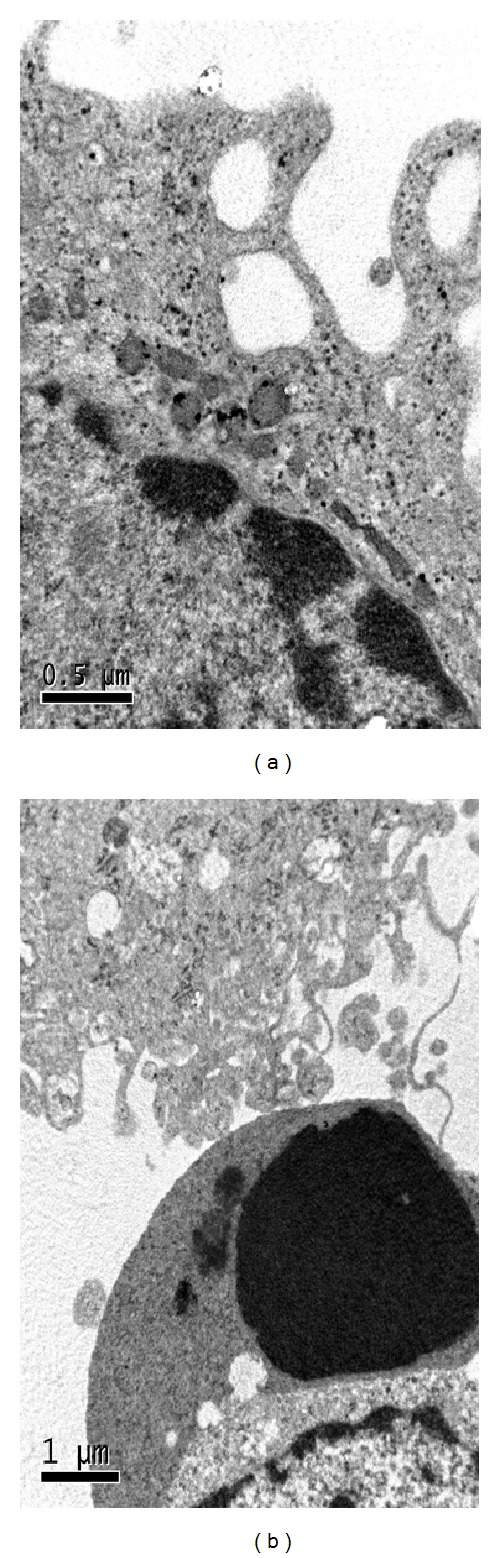
Apoptosis examined by TEM. (a) Striatal neuron with normal ultrastructure. (b) Typical apoptotic cell (intermediate-late stage) with shrunken nucleus and condensed chromatin.

Striatal neurons exposed to 30 mM GA for 24 h showed characteristic condensation of nuclear chromatin (Figure 8(b)). A normal neuron was shown in Figure 8(a) for comparison.

### 3.6. Effect of Glutaric Acid on Caspase 3 Transcript and Protein Levels

**Figure 9:**
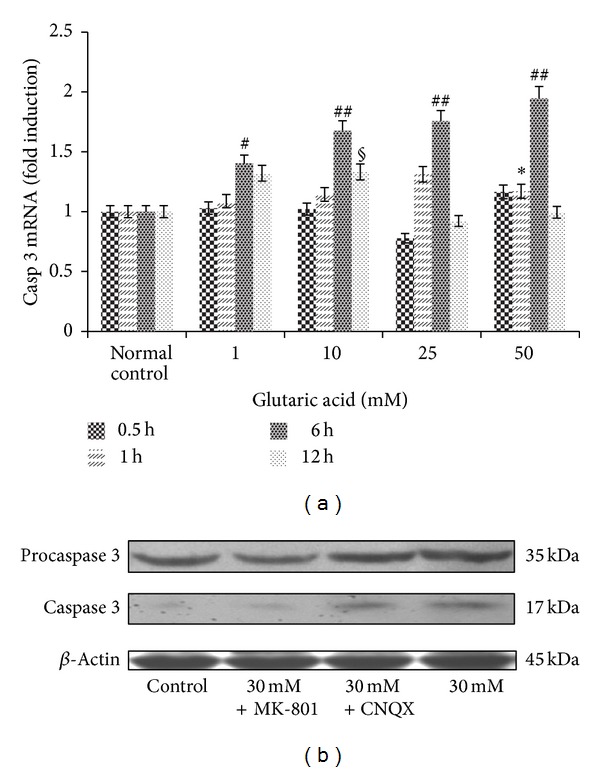
Changes in the expression of caspase 3. (a) Kinetics of caspase 3 mRNA expression. Note: cells treated with 1, 10, 25, and 50 mM GA were compared with normal control cells incubated for the same treatment periods, respectively. *P < 0.05 and **P < 0.01, compared to normal control cells incubated 1 h; ^#^ *P* < 0.05 and ^##^ *P* < 0.01, compared to normal control cells incubated 6 h; ^§^ *P* < 0.05 and ^§§^ *P* < 0.01, compared to normal control cells incubated 12 h. (b) Changes in the expression of caspase 3 proteins in striatal neurons treated with 30 mM GA. Primary cultures were exposed to 30 mM GA with or without antagonist preincubation for 24 h. Caspase 3 (precursor and active fragment) levels increased after GA incubation. The increase in active fragment of caspase 3 was partly reduced by addition of MK-801 but not CNQX.

Quantitative RT-PCR was performed to monitor mRNA expression of the apoptotic executioner caspase 3 (Figure 9(a)). The comparative 2^−ΔΔCt^ method was used to analyse relative expression levels. Caspase 3 mRNA expression at 6 hours after treatment with 1, 10, 25, and 50 mM GA was upregulated about 1.40-fold, 1.67-fold, and 1.95-fold, respectively, compared to control. Thus GA might induce apoptosis via caspase 3 activation.

Western blot analysis of caspase 3 (Figure 9(b)) indicated that 30 mM GA increased levels of the precursor protein and active fragments of caspase 3 (Mr 35 and 17 kDa protein band) relative to the normal control. MK-801 but not CNQX lowered the GA-induced active caspase 3 changes in protein level, which suggested that MK-801 at least partly protected the striatal neuronal cells against GA-induced apoptosis.

## 4. Discussion

Glutaric aciduria type I is an autosomal recessive disorder characterized by high levels of GA, 3-hydroxyglutaric acid (3-OHGA), glutaconic acid, and glutaryl-CoA in body fluids as well as degenerative changes in the striatal and frontotemporal cortical neurons. A deficiency of cerebral GCDH activity is attributed to the development of neurological damage in GA I patients. However, the comprehension of the degeneration mechanism in the basal ganglia still remains partial.

Three main mechanisms involved in the metabolites-mediated neuronal damage have been drawn from* in vitro* and* in vivo* studies: excitotoxicity, impairment of energy metabolism via decreasing of the activities of Na^+^/K^+^-ATPase and phosphocreatine in brain as well as inhibition of the tricarboxylic acid cycle, the different complexes of mitochondrial respiratory and the dicarboxylic acid shuttle between astrocytes and neurons [11, 12], and oxidative stress [19]. In addition, the limited efflux of BBB plays a central role in the neuropathogenesis: pathogenic GA and its metabolites were produced* de novo* inside the mitochondrial matrix and were trapped intracerebrally [10], so the pathologic events began in the neuronal compartment. But the cytotoxic effects are not necessarily restricted to neuronal mitochondrion. The kinetics of the metabolites transported through intra- and intercellular membranes and finally excreted into urine or faeces also seems to participate in the neuropathogenesis [20–22]. The destruction of striatal neurons is irreversible during the time window from birth to 36 months of age. Thus, the aim of the study is to elucidate the pathophysiology of striatal injury and the possible cell death pathway of the striatal neurons in GA I.

As seen in our study, GA concentration- and time-dependently induced mitochondrial dysfunction. In the MTT test, both 1 mM GA for ≥72 h incubation and higher concentration for a shorter period incubation were neurotoxic. However, 20 mM GA for ≥72 h incubation increased the percentage of apoptotic cells in flow cytometric analysis. The differences in GA concentration and incubation time of the detectable effects between MTT and flow cytometry suggests that mitochondrial dysfunction occurred apparently before structural change.

This* in vitro* study discloses that caspase-dependent apoptotic pathway was involved in GA-induced striatal neuronal apoptosis in striatal damage of GA I.

Supraphysiological concentrations of GA applied here are also higher than those used in most of other studies [4, 23]. Several factors should be taken into consideration: firstly, the pathogenic GA is produced within the mitochondria of cerebral neurons [10], where they reach their highest concentration before being transported to extracellular space. Moreover, the basal concentrations of GA in brains of affected patients and* Gcdh* ^−*/*−^ mice were calculated in the ranges of 0.5–5 mmol/L [2] and the level of GA further increased up to 10-fold during encephalopathic crises in mice [24], but there is no data on intracellular levels of specific brain regions in patients at such times. In summary, extracellular administration of GA not representing intracellular increase made it impossible to adopt the basal levels GA in plasma and urine to reproduce the physiological process. Indeed, there are several additional transport steps to reach the place generated* de novo in vivo* through astrocytes, across neural membranes, and finally through inner mitochondrial membranes [2]. Secondly, the primary striatal neurons with normal GCDH activity make the pathophysiological relevance of these works uncertain. The expression of GCDH is limited to neurons. Succinate-hydroxymethylglutarate CoA-transferase C7orf10 encoded and GCDH are both located in the mitochondria. C7orf10 can convert glutarate to glutaryl-CoA and GCDH catalyzes the oxidative decarboxylation of glutaryl-CoA to crotonyl-CoA and CO_2_. We postulate that extracellular GA administered was partly transferred into mitochondria and then degraded by succinate-hydroxymethylglutarate CoA-transferase and GCDH to crotonyl-CoA and CO_2_, alleviating the metabolic neurotoxicity of GA to some extent as physiological detoxification pathways [25]. Many reactions in the cerebral lysine-tryptophan degradation pathway* in vivo *are poorly understood still [26]. Thirdly, single dose or administration at two time points [16] were available in previous studies and this one, whereas in* in vivo* situation, neurons were chronically exposed to massive, sustained cerebral GA-related metabolites starting in utero and extending to the end of life [27, 28]. Since GA induced neuronal injury depending on the concentration and time of incubation, it is necessary to keep exposure to GA for enough time. However, the characteristics of primary cultured neurons limited the long-term research on GA and the other metabolites. Finally, many biochemical byproducts from lysine-tryptophan degradation, such as GA, 3-OHGA, GC, glutaryl-CoA, and quinolinic acid, are generated and accumulated in brain. It is difficult to discern their relative importance to neurotoxicity in GA 1 children. GA is the main metabolite accumulating in brain, which plays the major role in the inhibition of NaC3-mediated anaplerotic supply from astrocytes to neurons. However, some experiment proved that 3-hydroxyglutarate is the most toxic metabolite [16]. Comparing GA I with GA III, glutaryl-CoA or one of its downstream derivatives is likely to be the primary neurotoxin in GA I [25]. Also, the primary neuron model cannot substitute for model of cellular interaction participating in neurotoxicity (e.g., neuron, glial, vascular endothelial cells, etc.) [29]. Some studies suggested astrocyte proliferation protects neurons from the excitotoxic damage induced by 3-OHGA [30]. However, other experiments proved that astrocytic proliferation triggers progressive striatal degeneration in GA I [12, 31] by interrupting the anaplerotic supply of astrocytes and then failing to maintain the energy metabolism and synthesizing the neurotransmitters of glutamate and GABA in neurons. Disturbance of cerebral hemodynamics due to destroyed integrity of endothelial barriers also contributes to brain damage [22, 32]. In summary, synergistic effects among the metabolites [15] and all kinds of cellular dysfunctions perhaps could explain low concentrations of each and could combine to produce striatal degeneration in GA I children.

Caspase 3 participates in caspase-dependent apoptotic pathway and is the major effecter caspase in apoptotic cell death [33]. In this study, the upregulation in expressions of caspase 3 reveals that the apoptosis induced by GA might be at least partially caspase 3 dependent. The detection of caspases 8 and 9 and inhibitory interventions (Z-VAD-fmk, inhibition of caspases 3 and 9) should be conducted to identify the definite death pathway and ascertain the protection against GA-induced neuronal damage.

GA, structurally similar to excitotoxic amino acid glutamate, may cause direct interactions with NMDA receptors and indirect excitotoxicity by an imbalance between inhibitory and excitatory neurotransmission. The variable glutamate receptors expression profile may explain temporal and spatial vulnerability to GA I [34]. The conclusions drawn from previous studies on the involvement of glutamate receptors in GA I excitotoxic damage have been conflicting, which was summarized by Paris Jafari [4]. The main contradictions focus on if and which of quinolinic acid, 3-OHGA, or GA excited NMDA receptors or produced a synergistic excitation [35, 36], as well as whether NMDA receptor antagonists or non-NMDA receptor antagonists inhibited the neurotoxic effects of GA and 3-OHGA [37]. Discrepancy in results among the studies may be explained by differences in the experimental models, conditions, and approaches. In particular, the time of initial exposure to GA, which is closely related to NMDA receptors maturation [38], and the doses of GA used are critical. Lagranha et al. proved that glutamate receptor expression is higher in* Gcdh* ^−*/*−^ mice and that it may be involved in the pathophysiology of GA I and the vulnerability of striatum to injury in GA I patients [39]. In our research, we performed on the DIV 10 primary neuronal cultures with functional NMDA receptor; MK801, rather than CNQX, showed a partly protecting effect on the GA induced excitotoxicity, so apart from excitotoxicity, there must be other factors involved in the GA I neurodegeneration.

In conclusion, since GA does not accumulate in the extracellular space at the concentrations used in the “chemical” neuronal model which exhibiting enzymatically active endogenous GCDH, we should take caution to interpret these results in GA I patients, but it also can suggest some details of the mechanisms and enlighten the further research and possible therapeutic target for GA I.

## Figures and Tables

**Table 1:** Sequence of the primer pairs used in quantitative RT-PCR to assess expression of apoptotic genes.

Name	GenBank accession	Primer sequence (5′-3′)		Size (bp)
Caspase 3	NM_012922	Sense antisense	GATGTCGATGCAGCTAACC TGTCTCAATACCGCAGTCC	321

GAPDH	NM_017008	Sense antisense	GGCAAGTTCAACGGCACAG CGCCAGTAGACTCCACGACA	142
